# Blood lead level: an overlooked risk of carpal tunnel syndrome in hemodialysis patients

**DOI:** 10.1080/0886022X.2019.1657894

**Published:** 2019-09-09

**Authors:** Wen-Hung Huang, Ching-Chih Hu, Tzung-Hai Yen, Ching-Wei Hsu, Cheng-Hao Weng

**Affiliations:** a Kidney Research Center, Department of Nephrology, Linkou Chang Gung Memorial Hospital, Taoyuan, Taiwan;; b Clinical Poison Center, Linkou Chang Gung Memorial Hospital, Taoyuan, Taiwan;; c Chang Gung University College of Medicine, Taoyuan, Taiwan;; d Graduate Institute of Clinical Medical Sciences, College of Medicine, Chang Gung University, Taoyuan, Taiwan;; e Department of Hepatogastroenterology and Liver Research Unit, Chang Gung Memorial Hospital, Keelung, Taiwan

**Keywords:** Carpal tunnel syndrome, hemodialysis, lead, normalized protein catabolic rate

## Abstract

**Introduction:** Carpal tunnel syndrome (CTS) is a severe complication observed in long-term maintenance hemodialysis (MHD) patients. The most common cause of CTS is dialysis-related β2-microglobulin amyloidosis, which is associated with inflammation and oxidative stress in dialysis patients. Patients on MHD have higher blood lead levels (BLLs) than the general population. Lead (Pb) exposure in chronic dialysis patients has been noted to induce oxidative stress and inflammation. Therefore, lead-related inflammation and oxidative stress might contribute to CTS.

**Methods:** The medical records of 866 MHD patients were reviewed. Two hundred and thirty-four patients with symptoms of CTS were surveyed by senior neurologists via physical examinations and nerve conduction studies. Patients in this study were stratified into groups with low-normal (<10 μg/dL), high-normal (10 to 20 μg/dL), and abnormal (>20 μg/dL) BLLs. The associations between CTS and BLLs and the clinical data were analyzed.

**Results:** Multivariate logistic regression analyses showed that Log BLL (OR: 54.810, 95% CI: 13.622–220.54, *p* < .001), high-normal BLLs (OR: 4.839, 95% CI: 2.262–10.351, *p* < .001) with low-normal BLL as a reference, high BLLs (OR: 12.952, 95% CI: 5.391–31.119, *p* < .001) with low-normal BLL as a reference, and a BLL >12.3 μg/dL (OR: 6.827, 95% CI: 3.737–12.472, *p* < .001) were positively associated with CTS according to three different analyses.

**Discussion:** In conclusion, blood lead levels were positively associated with CTS in patients on MHD. Dialysis patients should pay more attention to their environmental exposure to Pb. Avoidance of environmental Pb may reduce the incidence of CTS in MHD patients. Future studies will address the role of Pb in the pathophysiology of CTS in this patient population.

## Introduction

Carpal tunnel syndrome (CTS) is a severe complication found in long-term maintenance hemodialysis (MHD) patients [1]. The most common cause of CTS is the deposition of dialysis-related β2-microglobulin amyloidosis in the carpal tunnel [2]. β2-microglobulin has been noted to be associated with systemic inflammation and oxidative stress in dialysis patients [3]. The other etiologies of CTS in MHD patients include hemodialysis (HD) fistulas or grafts, increased venous pressure during HD, bleeding from the vascular access, steal syndrome and compression due to calcium phosphate deposits [4–6]. Lead (Pb) has been noted to accumulate in MHD patients [7,8]. Patients on MHD have higher blood lead levels (BLLs) than the general population in Taiwan [9]. Since our HD centers have very low lead levels (< 2 μg/L) in the dialysis reverse osmosis water and dialysate, the elevated BLLs observed in MHD patients might result from a complete loss of renal function to excrete lead from the body. In addition, hemodialysis results in a difficulty of removing lead from the body [10]. Therefore, the elevation of BLLs in MHD patients might result from environmental exposure. Environmental sources of lead include petroleum, industrial production, paint, water pipes made of lead and food stored in cans [11–14]. Mortality in dialysis patients is significantly associated with high BLLs [9,15]. Lead exposure in MHD patients has been noted to induce oxidative stress and inflammation [16,17]. Therefore, lead-related inflammation and oxidative stress might contribute to CTS. There has been no previous study that has identified a correlation between BLLs and CTS in MHD patients.

## Materials and methods

### Compliance with ethical standards

#### Ethical approval

This study was approved by the Medical Ethics Committee of Chang Gung Memorial Hospital Linkou branch (Institutional Review Board approval number: 101-5199B), a tertiary referral center located in the northern part of Taiwan. This study was carried out in accordance with The Code of Ethics of the World Medical Association (Declaration of Helsinki).

### Informed consent

The Medical Ethics Committee of Chang Gung Memorial Hospital Linkou branch gave the approval that informed consent was not necessary in this retrospective study.

### Patients

We reviewed the medical records of HD patients from the three HD centers of LinKou, Taipei, and Taoyuan Chang Gung Memorial Hospitals. Patients who were 18-year old or older and who had received HD for more than 6 months were recruited in this study. Patients with cancers or active infectious diseases and those who had been admitted or had received surgeries within 3 months of the investigation were excluded. Most patients received 4 h of HD, 3 times a week. Eight hundred and sixty-six MHD patients were included, and these 866 patients included every patient in the 3 HD units, minus the excluded patients. Seventy-three patients underwent dialysis for 4 h, 2 times a week, and 793 patients underwent dialysis for 4 h, 3 times a week. One hundred and eighty-seven patients received hemodiafiltration (HDF). The reasons for receiving HDF in these patients included hyperphosphatemia, malnutrition, insomnia, restless-leg syndrome, polyneuropathy, anemia, pruritus, and dialysis-associated amyloidosis. Hemodialysis was performed using single-use hollow-fiber dialyzers equipped with modified cellulose, polyamide, or polysulfone membranes. The dialysate used in all patients had a standard ionic composition with a bicarbonate-based buffer. The dialysate compositions were as follows: sodium concentration: 140 mEq/L; potassium concentration: 2.0 mEq/L; bicarbonate concentration: 35 mEq/L; and calcium concentration: 2.5–3.5 mEq/L. The blood flow rate ranged from 200 to 380 mL/min. The dialysate flow rate was 500 mL/min. The incidence of cardiovascular diseases (CVDs) including cerebrovascular disease, coronary artery disease, congestive heart failure, and peripheral vascular disease was recorded. Board-certified senior neurologists with ≥10 years’ experience diagnosed the presence of CTS via physical examinations and nerve conduction studies, and they were blinded to the BLLs.

### Criteria for the diagnosis of CTS

Symptoms: Pain or paresthesia (numbness and tingling) in a distribution that included the median nerve territory, with involvement of the first three digits and the radial half of the fourth digit.

Physical findings: The physical examination findings for a diagnosis of CTS included Phalen’s and Tinel’s signs and hand elevation tests.

Nerve conduction study (NCS) results: measurement of conduction velocity across the carpal tunnel, as well as determination of the amplitude of sensory and motor responses. Carpal tunnel syndrome results in local conduction block and/or slowing of motor and sensory conduction across the wrist. Sensory fibers seem to be more sensitive to compression than motor fibers. As a result, sensory fibers typically demonstrate changes in NCSs earlier than motor fibers.

The CTS diagnosis was unilateral. Fifty-four patients had CTS on their access arm, and 22 patients had CTS on their non-access arm. We excluded patients who developed CTS that was previously corrected with surgery because we did not have the BLLs at the time they received surgery, and any BLLs measured after surgery may not have been related to their CTS.

### Measurement of BLLs

Blood lead levels were measured from the arterial side of the vascular access immediately before the initiation of HD after a 2-day interval without HD by a previously described method with electrothermal atomic-absorption spectrometry (SpectrAA-200Z; Agilent Technologies, www.agilent.com) [18,19].

Definition of low-normal, high-normal and high blood lead levels

We stratified the MHD patients into three groups of low-normal: <10 μg/dL, high-normal: 10 to 20 μg/dL, and abnormal: >20 μg/dL [10] BLLs according to the medical records.

### Laboratory parameters

All blood samples were obtained from the arterial side of the vascular access immediately before the initiation of HD after a 2-day interval without HD. Biochemical data were measured with standard laboratory protocols. The adequacy of hemodialysis was calculated by the Daugirdas method and was expressed as Kt/*V*_urea_. The KT/*V*_urea_ in our HD units is a single pool. Anuria was defined as a daily urine volume <100 mL. Non-anuria was defined as a daily urine volume ≥100 mL.

### Statistical analysis

The normal distribution of variables was analyzed by the Kolmogorov–Smirnov test. Normal distribution was defined as a *p* values >.05. Continuous variables are expressed as the mean ± standard deviation/median (interquartile range), and categorical variables are expressed as numbers and percentages. Comparisons between the three groups were performed using the analysis of variance (ANOVA). Log-transformed Pb and intact parathyroid hormone (iPTH) levels were used for analysis. Predictors for CTS were analyzed by univariate and multivariate logistic regression analyses. Calibration was analyzed by the Hosmer–Lemeshow goodness-of-fit test to compare the number of observed and predicted CTS cases in risk groups for the entire range of CTS probabilities. Discrimination was analyzed by the area under the receiver operating characteristic curve (AUROC). Cutoff values as well as the sensitivity, specificity, and overall correctness were calculated according to the AUROC. Finally, the cutoff points were calculated by acquiring the best Youden index (sensitivity + specificity–1). Data were analyzed using SPSS, version 12.0 for Windows 95 (SPSS Inc., Chicago, IL, USA). The level of significance was set at *p* < .05.

## Results

### Study population characteristics

Eight hundred and sixty-six MHD patients were included. The patients were 56.18- ± 13.59-year old, and 440 patients were male (50.8%). The average HD duration was 6.96 ± 5.35 years. After reviewing the medical records of these 866 MHD patients, we found that 234 patients with CTS symptoms visited neurology clinics and were surveyed by senior neurologists. Senior neurologists then performed physical examinations and arranged an NCS for the definite diagnosis of CTS. Seventy-six patients (8.8%) had CTS. The average BLL was 10.39 μg/dL (7.26 μg/dL, 14.19 μg/dL; Table 1). We divided patients into low-normal BLL, high-normal BLL, and high BLL groups. Patients with high BLLs demonstrated a significant trend for a higher percentage of patients of the male sex (*p* = .09); significantly higher instances of hepatitis C infection (HCV; *p* < .001), CTS (*p* < .001), and receiving hemodiafiltration (*p* < .001); a lower percentage of DM (*p* < .001) and residual daily urine of >100 mL (*p* = .035); a higher hemodialysis duration (*p* < .001), Kt/*V*_urea_ (Daugirdas; *p* < .001), and hemoglobin (Hb) and iPTH levels; and lower erythropoietin dosages (*p* < .001) and serum albumin and serum ferritin levels (*p* = .001; Table 2).

**Table 1.** Baseline characteristics of 866 MHD patients.

Characteristics	Total (*n* = 866) Mean ± SD/ Median (interquartile range)	Normal range in CGMH
*Demographics*		
Age (y)	56.18 ± 13.59	
Male, *n* (%)	440 (50.8%)	
Body mass index (kg/m^2^)	22.19 ± 3.18	
Smoking, *n* (%)	150 (17.3%)	
*Co-morbidity*		
Diabetes mellitus, *n* (%)	192 (22.2%)	
Hypertension, *n* (%)	339 (39.1%)	
Previous CVD, *n* (%)	41 (4.7%)	
HBV, *n* (%)	98 (11.3%)	
HCV, *n* (%)	168 (19.4%)	
CTS, *n* (%)	76 (8.8%)	
*Dialysis Related Data*		
Hemodialysis duration (y)	6.96 ± 5.35	
Erythropoietin (U/kg/week)	73.62 ± 47.37	
Fistula as blood access, *n* (%)	689 (79.6%)	
Hemodiafiltration, *n* (%)	187 (21.6%)	
Kt/*V*_urea_ (Daugirdes)	1.79 ± 0.32	
nPCR (g/kg/day)	1.18 ± 0.26	
Residual daily urine of >100 mL, *n* (%)	178 (20.6%)	
*Biochemical Data*		
Hemoglobin (g/dL)	10.51 ± 1.36	M: 13.5–17.5 F: 12–16
Albumin (g/dL)	4.06 ± 0.34	3.5–5.5
Creatinine (mg/dL)	10.88 ± 2.39	M: 0.64–1.27 F: 0.44–1.03
Ferritin (μg/L)^a^	305.0 (129.57, 504.45)	M: 22–322 F: 10–291
Corrected-calcium (mg/dL)	9.94 ± 0.93	7.9–9.9
Phosphate (mg/dL)	4.84 ± 1.35	2.4–4.7
Intact parathyroid hormone (pg/mL)^a^	130.1 (52.52, 319.2)	ESRD: 168–342
hsCRP (mg/L)^a^	2.95 (1.4, 7.01)	<5
hsCRP > 3 mg/L, *n* (%)	420 (48.5%)	
*Cardiovascular Risks*		
Cholesterol (mg/dL)	171.3 ± 37.66	<200
Triglyceride (mg/dL)	164.33 ± 115.8	<150
LDL (mg/dL)	94.83 ± 30.59	<100
BLL (ug/dL)^a^	10.39 (7.26, 14.19)	<10

M: male; F: female; ESRD: end stage renal disease; MHD: maintenance hemodialysis; CTS: carpal tunnel syndrome; CVD: cardiovascular disease; HBV: hepatitis B virus infection; HCV: hepatitis C virus infection; nPCR: normalized protein catabolic rate; hsCRP: high-sensitivity C-reactive protein; LDL: low density lipoprotein; Kt/*V*_urea_ (Daugirdes): dialysis clearance of urea; BLL: blood lead level; CGMH: Chang Gung Memorial Hospital.

a Non-normal distribution data are presented as median (interquartile range).

**Table 2.** Comparison of the baseline characteristics between different BLLs.

	Low-normal BLL (*n* = 408)	High-normal BLL (*n* = 380)	High BLL (*n* = 78)	*p* Value
Age (y)	56.21 ± 13.89	55.87 ± 13.26	57.52 ± 13.66	.72
Male, *n* (%)	48.3	52.1	57.7	.09
Body mass index (kg/m^2^)	22.21 ± 3.27	22.22 ± 3.13	21.94 ± 3.01	.64
Smoking, *n* (%)	15.7	18.2	21.8	.15
Diabetes mellitus, *n* (%)	27.5	19.2	9.0	<.001
Hypertension, *n* (%)	39.0	39.2	39.7	.89
Previous CVD, *n* (%)	4.2	6.1	1.3	.92
HBV, *n* (%)	10.0	12.1	14.1	.22
HCV, *n* (%)	14.7	22.1	30.8	<.001
CTS, *n* (%)	2.2	11.6	29.5	<.001
Hemodialysis duration (y)	5.73 ± 4.75	7.58 ± 5.39	10.28 ± 6.18	<.001
Erythropoietin (U/kg/week)	79.58 ± 46.34	69.57 ± 47.73	62.27 ± 47.33	<.001
Fistula as blood access, *n* (%)	78.7	79.7	83.3	.39
Hemodiafiltration, *n* (%)	15.0	24.7	41	<.001
Kt/*V*_urea_ (Daugirdes)	1.75 ± 0.30	1.82 ± 0.32	1.90 ± 0.39	<.001
nPCR (g/kg/day)	1.18 ± 0.26	1.19 ± 0.27	1.16 ± 0.27	.97
Residual daily urine of >100 mL, *n* (%)	23.5	18.4	15.4	.035
Hemoglobin (g/dL)	10.33 ± 1.31	10.63 ± 1.34	10.88 ± 1.57	<.001
Albumin (g/dL)	4.08 ± 0.33	4.05 ± 0.35	3.98 ± 0.36	.018
Creatinine (mg/dL)	10.88 ± 2.46	10.90 ± 2.29	10.79 ± 2.47	.88
Corrected-calcium (mg/dL)	9.89 ± 0.90	9.96 ± 0.94	10.06 ± 0.99	.11
Phosphate (mg/dL)	4.83 ± 1.35	4.87 ± 1.37	4.72 ± 1.26	.76
Ferritin (μg/L)^a^	359.9 (165.1,569.85)	272.4 (111.25,445.55)	283.55 (156.32,420.45)	.001
Intact parathyroid hormone (pg/mL)^a^	112.4 (41.5,249.1)	144.25 (55.6,365.42)	195.85 (82.67,435.27)	<.001
hsCRP (mg/L)^a^	2.87 (1.34,7.51)	2.98 (1.54,6.49)	3.36 (1.4,7.38)	.6
LDL (mg/dL)	93.95 ± 30.63	95.94 ± 30.57	93.90 ± 30.69	.62

Low-normal BLL, BLL <10 μg/dL (reference).

High-normal BLL, 20 μg/dL > BLL ≥10 μg/dL.

High BLL, BLL ≥20 μg/dL.

BLL: blood lead level; MHD: maintenance hemodialysis; CTS: carpal tunnel syndrome; CVD: cardiovascular disease; HBV: hepatitis B virus infection; HCV: hepatitis C virus infection; nPCR: normalized protein catabolic rate; hsCRP: high-sensitivity C-reactive protein; LDL: low density lipoprotein; Kt/*V*_urea_ (Daugirdes): dialysis clearance of urea.

a Non-normal distribution data are presented as median (interquartile range).

### Predictors of carpal tunnel syndrome according to univariate logistic regression

Univariate logistic regression analyses showed that HCV, HD duration, Kt/*V*_urea_ (Daugirdas), normalized catabolic rate (nPCR), Hb level, hypoalbuminemia (albumin <4 g/dL), log iPTH level, and BLLs were positively associated with CTS (Table 3). Diabetes mellitus and non-anuria were negatively associated with CTS.

**Table 3.** Univariate logistic regression analysis of predictors of CTS in MHD Patients.

Variables	Odds ratio (OR) 95% confidence Intervals (CI)	*p*
Age (years)	1.007 (0.99, 1.025)	.432
Male	0.812 (0.5, 1.30)	.386
Body mass index (kg/m^2^)	0.96 (0.89, 1.03)	.33
Smoking	0.7 (0.35, 1.4)	.31
Diabetes mellitus	0.33 (0.15, 0.73)	.007
Hypertension	0.95 (0.58, 1.55)	.85
Previous CVD	0.52 (0.12, 2.19)	.37
HBV	0.65 (0.27, 1.53)	.32
HCV	2.07 (1.23, 3.47)	.006
Hemodialysis duration (years)	1.17 (1.13, 1.22)	<.001
Fistula as blood access	1.76 (0.89, 3.51)	.1
Hemodiafiltration	1.54 (0.91, 2.6)	.11
Kt/*V*_urea_ (Daugirdes)	3.39 (1.71, 6.7)	<.001
nPCR (g/kg/day)	3.17 (1.35, 7.4)	.008
Non-Anuria	0.3 (0.13, 0.72)	.007
Hemoglobin (g/dL)	1.21 (1.02, 1.43)	.027
Albumin < 4 g/dL	2.038 (1.26, 3.27)	.003
Albumin	0.489 (0.253, 0.946)	.034
Creatinine (mg/dL)	0.94 (0.85, 1.04)	.3
Corrected-calcium (mg/dL)	1.18 (0.92, 1.51)	.18
Phosphate (mg/dL)	0.95 (0.79, 1.13)	.57
Log ferritin	1.07 (0.65, 1.78)	.76
Log iPTH	2.62 (1.63, 4.22)	<.001
hsCRP >3.0 mg/L	1.18 (0.73, 1.90)	.48
Cholesterol (mg/dL)	1.002 (0.99, 1.008)	.63
Triglyceride (mg/dL)	1.00 (0.99, 1.002)	.73
Log BLL	118.67 (31.72, 443.92)	<.001
*BLLs*		<.001
Low-normal BLL, BLL < 10 ug/dL (reference)		
High-normal BLL, 20 ug/dL > BLL ≥ 10 ug/dL	5.806 (2.79, 12.06)	<.001
High BLL, BLL ≥ 20 ug/dL	18.539 (8.16, 42.11)	<.001
Mean previous 12-month Environmental PM_2.5_ (ug/m^3^)	1.078 (1.008, 1.153)	.027

CTS: carpal tunnel syndrome; MHD: maintenance hemodialysis; CVD: cardiovascular disease; HBV: hepatitis B virus infection; HCV: hepatitis C virus infection; nPCR: normalized protein catabolic rate; hsCRP: high-sensitivity C-reactive protein; LDL: low density lipoprotein; Kt/*V*_urea_ (Daugirdes): dialysis clearance of urea; iPTH: intact parathyroid hormone; Pb: blood lead; BLL: blood lead level; PM_2.5_: particulate matter with an aerodynamic diameter of <2.5 mm.

### Predictors of carpal tunnel syndrome according to multivariate logistic regression

Univariate logistic regression analyses demonstrated that BLLs, high-normal BLLs and high BLLs were positive predictors of CTS with low-normal BLLs as a reference. Particulate matter with an aerodynamic diameter of <2.5 mm (PM_2.5_) was also a positive predictor of CTS, as found in our previous study [20] (Table 3). Multivariate logistic regression analysis included BLLs, high-normal BLLs and high BLLs independently as well as those with *p* < .05 in univariate logistic regression analysis. The following analyses of A, B, and C were identical to each other except for the nature of the BLLs (log Pb vs. 3 BLL ranges vs. BLL cutoff point). Analysis A showed that HD duration [odds ratio (OR): 1.195, 95% confidence interval (CI): 1.131–1.264, *p* < .001], nPCR (OR: 3.734, 95% CI: 1.478–9.482, *p* = .005), hypoalbuminemia (albumin <4 g/dL; OR: 2.102, 95% CI: 1.220–3.621, *p* = .007), and Log BLL (OR: 54.810, 95% CI: 13.622–220.54, *p* < .001) were positively associated with CTS (Table 4). Analysis B showed that HD duration (OR: 1.197, 95% CI: 1.132–1.265, *p* < .001), nPCR (OR: 3.862, 95% CI: 1.530–9.751, *p* = .004), hypoalbuminemia (albumin <4 g/dL; OR: 2.187, 95% CI: 1.269–3.768, *p* = .005), high-normal BLLs (OR: 4.839, 95% CI: 2.262–10.351, *p* < .001) with low-normal BLL as a reference, and high BLLs (OR: 12.952, 95% CI: 5.391–31.119, *p* < .001) with low-normal BLL as a reference were positively associated with CTS (Table 5). The discrimination power of BLLs for predicting CTS was an AUROC ± SE: 0.775 ± 0.027 with *p* < .001. The cutoff point of BLLs for predicting CTS was 12.3 μg/dL with a sensitivity of 0.79 and a specificity of 0.69. Analysis C showed that HD duration (OR: 1.203, 95% CI: 1.138–1.273, *p* < .001), nPCR (OR: 3.207, 95% CI: 1.273–8.078, *p* = .013), hypoalbuminemia (albumin <4 g/dL; OR: 2.221, 95% CI: 1.284–3.842, *p* = .004), and BLL >12.3 μg/dL (OR: 6.827, 95% CI: 3.737–12.472, *p* < .001) were positively associated with CTS (Table 6). Unlike our previous study, PM_2.5_ was not a predictor of CTS according to these multivariate logistic regression analyses. Based on the above analyses, a number of factors were significant predictors of CTS. However, high BLL (in the three different analyses) was a much more powerful predictor than any of the other factors.

**Table 4.** Analysis A: Multivariate logistic regression analysis of predictors of CTS in MHD Patients with log Pb.

Variables	Odds ratio (OR) 95% confidence Intervals (CI)	*p*
Diabetes mellitus	0.983 (0.931–1.034)	NS
HCV	1.482 (0.911, 2.192)	NS
Hemodialysis duration (years)	1.195 (1.131,1.264)	<.001
Kt/*V*_urea_ (Daugirdes)	2.874 (0.951–4.692)	NS
nPCR (g/kg/day)	3.743 (1.478,9.482)	.005
Non-Anuria	0.453 (1.035–0.722)	NS
Hemoglobin (g/dL)	1.070 (0.953–1.211)	NS
Albumin < 4 g/dL	2.102 (1.220,3.621)	.007
Log iPTH	1.514 (0.955–2.026)	NS
Log BLL	54.810 (13.622,220.54)	<.001
Mean previous 12-month Environmental PM_2.5_ (ug/m^3^)	1.073 (0.999,1.152)	NS

CTS: carpal tunnel syndrome; MHD: maintenance hemodialysis; nPCR: normalized protein catabolic rate; hsCRP: high-sensitivity C-reactive protein; Kt/*V*_urea_ (Daugirdes): dialysis clearance of urea; iPTH: intact parathyroid hormone; BLL: blood lead level; PM_2.5_: particulate matter with an aerodynamic diameter of <2.5 mm.

**Table 5.** Analysis B: Multivariate logistic regression analysis of predictors of CTS in MHD Patients with BLL category.

Variables	Odds ratio (OR) 95% confidence Intervals (CI)	*p*
Diabetes mellitus	0.972 (0.946–1.041)	NS
HCV	1.373 (0.925, 1.996)	NS
Hemodialysis duration (years)	1.197 (1.132, 1.265)	<.001
Kt/*V*_urea_ (Daugirdes)	2.913 (0.934–4.731)	NS
nPCR (g/kg/day)	3.862 (1.530, 9.751)	.004
Non-Anuria	0.452 (1.034–0.731)	NS
Hemoglobin (g/dL)	1.075 (0.964–1.221)	NS
Albumin < 4 g/dL	2.187 (1.269, 3.768)	.005
Log iPTH	1.512 (0.957–2.028)	NS
*BLLs*		<.001
Low-normal BLL, BLL <10 μg/dL (reference)		
High-normal BLL, 20 μg/dL > BLL ≥10 μg/dL	4.839 (2.262, 10.351)	<.001
High BLL, BLL ≥20 μg/dL	12.952 (5.391, 31.119)	<.001
Mean previous 12-month Environmental PM_2.5_ (ug/m^3^)	1.075 (0.996, 1.159)	NS

CTS: carpal tunnel syndrome; HCV: hepatitis C virus infection; MHD: maintenance hemodialysis; nPCR: normalized protein catabolic rate; Kt/*V*_urea_ (Daugirdes): dialysis clearance of urea; iPTH: intact parathyroid hormone; BLL: blood lead level; PM_2.5_: particulate matter with an aerodynamic diameter of <2.5 mm.

**Table 6.** Analysis C: Multivariate logistic regression analysis of predictors of CTS in MHD Patients with Pb ≥12.3 μg/dL.

Variables	Odds ratio (OR) 95% confidence intervals (CI)	*p*
Diabetes mellitus	0.991 (0.955–1.031)	NS
HCV	1.378 (0.929, 1.987)	NS
Hemodialysis duration (years)	1.203 (1.138, 1.273)	<.001
Kt/*V*_urea_ (Daugirdes)	2.918 (0.938–4.742)	NS
nPCR (g/kg/day)	3.207 (1.273, 8.078)	.013
Non-Anuria	0.460 (1.040–0.742)	NS
Hemoglobin (g/dL)	1.077 (0.975–1.223)	NS
Albumin <4 g/dL	2.221 (1.284, 3.842)	.004
Log iPTH	1.521 (0.957–2.036)	NS
BLL ≥12.3 μg/dL	6.827 (3.737, 12.472)	<.001
Mean previous 12-month Environmental PM_2.5_ (ug/m^3^)	1.009 (0.999, 1.020)	NS

CTS: carpal tunnel syndrome; HCV: hepatitis C virus infection; MHD: maintenance hemodialysis; nPCR: normalized protein catabolic rate; Kt/*V*_urea_ (Daugirdes): dialysis clearance of urea; iPTH: intact parathyroid hormone; BLL: blood lead level; PM_2.5_: particulate matter with an aerodynamic diameter of <2.5 mm.

## Discussion

In the present study, we showed that a high BLL, presumably from environmental exposure, predicted CTS in MHD patients. One of the etiologies of CTS in MHD patients is the presence of calcium phosphate deposits [4]. Lead has direct calcifying effects in connective tissue [21]. Therefore, lead may aggravate uremic tumoral calcinosis in MHD patients in the carpal tunnel and, thus, result in CTS.

Carpal tunnel syndrome in MHD patients has been well known to be caused by dialysis-related β2-microglobulin amyloid deposition in the carpal tunnel [20,22]. β2-microglobulin amyloid deposition induces inflammation with a significant recruitment of macrophages that express cytokines, such as interleukin-1, tumor necrosis factor-α (TNF-α), and transforming growth factor-β (TGF-β) [23]. Cheng and Liu showed that Pb increased lipopolysaccharide-induced liver damage via TNF-α and oxidative stress [24]. Lead damages cellular components via elevated levels of oxidative stress and ·OH free radicals via the Fenton reaction [25]. Huang et al. also demonstrated that increasing BLLs were associated with elevated TNF-α levels [26]. Lead might induce β2-microglobulin amyloid deposition in the carpal tunnel through the effect of TNF-α. After Pb exposure, the high expression of IL-1β, IL-6, and glial fibrillary acidic protein in the hippocampus of pups may contribute to neurotoxicity [27]. Lead also induces TGF-β expression in various organs [28–31]. All the above studies show that Pb might represent one of the etiologies of β2-microglobulin amyloid deposition in the carpal tunnel. The above studies all show indirect correlations between BLLs and β2-microglobulin amyloid deposition in the carpal tunnel. There have still been no studies that have demonstrated a direct association between blood or tissue Pb levels and β2-microglobulin amyloid in CTS surgical samples or autopsies.

Our study showed that a number of factors were significant predictors of CTS. However, the different nature of BLLs (in the three different analyses) was a much more powerful predictor than any of the other factors. High-normal BLLs and high BLLs were significantly associated with CTS compared with low-normal BLLs. The cutoff value of BLLs for predicting CTS in our study was 12.3 μg/dL. Lin et al. showed that in MHD patients, a high blood lead level (BLLs >12.64 μg/dL) was associated with increased hazard ratios for all-cause, cardiovascular-cause, and infection-cause 18-month mortality [9]. Although, our cutoff value of BLLs for CTS was lower than 12.64 μg/dL, BLLs above 12.3 μg/dL were still associated with CTS, which represents a severe complication found in MHD patients. Another study by Lin et al. also showed that basal high BLLs (>8.66 μg/dL; hazard ratio [HR] = 3.745, 95% confidence interval [95% CI] = 1.218–11.494, *p* = .001) and mid-level BLLs (5.62–8.66 μg/dL; HR = 1.867, 95% CI = 1.618–2.567, *p* = .001) were associated with increased HR for all-cause mortality in chronic peritoneal dialysis patients [15]. Therefore, the normal values of BLLs in MHD patients should be reinvestigated; otherwise, there might be no known normal or safe values of BLLs in MHD patients.

In Taiwan, a petrol-lead phase-out program was developed and divided into three main phases: (1) a leaded petrol phase (0.72–0.12 g/L; 1981–1992); (2) a transition phase (0.08 and 0.026 g/L; 1993–1999); and (3) a phrase that involved the ban of leaded petrol (after 2000). The mean blood lead levels decreased from 20.14 μg/dL in the leaded petrol phase to 3.00 μg/dL in the unleaded petrol phase in the general population [32], and this might be the reason that the median BLLs of our included MHD patients was 10.39 μg/dL, which was near the normal value of <10 μg/dL in our hospital. Pirkle et al. also showed that in the United States, the mean BLLs of persons aged 1 to 74 years decreased by 78%, from 12.8 to 2.8 μg/dL after the removal of 99.8% of lead from gasoline [33].

Our study showed that BLLs were the strongest predictor of CTS in MHD patients; however, blood generally carries only a small fraction of the total lead burden in the body. Heard and Chamberlain has demonstrated that the half-life of blood lead is approximately 15 days in adults and that it will be absorbed by bone and excreted in urine [34]. Therefore, BLLs reflect recent or ongoing exposure. Most of the Pb in the body is stored in soft tissues and bones [35]. Metabolic acidosis in patients with chronic kidney disease and end-stage kidney disease will cause the resorption of bone and the release of bone lead into the serum. BLLs might involve recent lead exposure in addition to the release of lead from bone. However, if MHD patients receive adequate dialysis, the metabolic acidosis status can be corrected, and the release of lead from bone can be minimized. Our study would be more powerful if tissue or blood lead levels after chelation were used for analysis. The major limitation of this study was that we did not acquire these data.

## Conclusion

In conclusion, blood lead levels were positively associated with CTS in patients on MHD in our study. Dialysis patients should pay more attention to their environmental exposure to Pb. Avoidance of environmental Pb may reduce the incidence of CTS in MHD patients. Future studies will address the role of Pb in the pathophysiology of CTS in this patient population.
